# Challenges in Tuberculosis Clinical Trials in the Face of the COVID-19 Pandemic: A Sponsor’s Perspective

**DOI:** 10.3390/tropicalmed5020086

**Published:** 2020-05-27

**Authors:** I.D. Rusen

**Affiliations:** Senior Vice President, Research Division, Vital Strategies, New York, NY 10005, USA; IRusen@vitalstrategies.org

**Keywords:** MDR-TB, clinical trials, COVID-19

## Abstract

The COVID-19 pandemic has caused unforeseen and extreme changes in societal and health system functioning not previously experienced in most countries in a lifetime. The impact of the pandemic on clinical trials can be especially profound given their complexities and operational requirements. The STREAM Clinical Trial is the largest trial for MDR-TB ever conducted. Currently operating in seven countries, the trial had 126 participants on treatment and 312 additional participants in active follow up as of March 31, 2020. Areas of particular concern during this global emergency include treatment continuity, supply chain management and participant safety monitoring. This commentary highlights some of the challenges faced due to the pandemic and the steps taken to protect the safety of trial participants and the integrity of the trial.

The COVID-19 pandemic has caused unforeseen and extreme changes in societal and health system functioning not previously experienced in most countries in a lifetime [1]. The disruptions have been dynamic-varying by time and geography-which adds to their unpredictable effect. The negative impact of COVID-19 on tuberculosis (TB) programs has recently been reported with models predicting 126,100 excess TB deaths over the next five years for every one month of COVID-related lockdown [2]. Clinical trial implementation is especially challenging in this pandemic environment, requiring sponsors to adhere to trial protocols and regulatory requirements as closely as possible while ensuring the safety of trial participants and staff.

Tuberculosis clinical trials carry inherent challenges at the best of times. Locations with the highest tuberculosis burden often have less resilient regulatory infrastructure, complex operational environments and more limited clinical trial experience [3]. During an unexpected and large-scale disruption like COVID-19, the impact of these weaknesses becomes more magnified.

STREAM is the largest multi-country trial for multidrug-resistant tuberculosis (MDR-TB) ever conducted. Conceived by the Union and global partners with initial funding from USAID, the trial has recruited over 1000 patients to two distinct stages. Results from STREAM Stage 1 were published in 2019 and 2020 [4,5], and recruitment was recently completed for the second stage in January, 2020. As of March 31, 2020, 126 participants remain on MDR-TB treatment with 21 of those in the intensive phase of treatment and 312 participants remain in active follow-up.

Stage 2 of STREAM is a registration trial [6], which adds complexity to implementation. It incorporates central safety and microbiology testing, requiring regular export of biological samples. In addition, a contract research organization (CRO) is employed to conduct onsite monitoring and source data verification to ensure data quality.

The COVID-19 pandemic has had an important impact on the implementation of STREAM. Key challenges faced by STREAM (and similar trials) together with the responses of the trial team to date, are outlined in this commentary.

Early in the COVID-19 outbreak, working with our main implementing partner, Medical Research Council Clinical Trials Unit at University College London, a STREAM-COVID-19 Task Force was formed to identify and address the risks and challenges arising due to the pandemic. A continuity plan was developed, and the task force met (and continues to meet) weekly to respond to the rapidly changing circumstances.

Enhanced communication with trial sites was initiated to provide timely guidance to the sites, and also ensure that the Task Force was aware of any pandemic-related issues faced by sites. Sites were provided educational material on TB and COVID-19 and were requested to inform the central trial team of any unforeseen challenges. Additional site resources were considered upon request, such as office space away from health care facilities. Importantly, there were no reported shortages of personal protective equipment (PPE). Sites have also been directed to maintain regular communication with all trial participants. Participants are to be informed of potential changes to their follow-up schedules, as well as other site-specific changes. Importantly all participants were reassured about their care during the trial and instructed to raise any concerns with the trial team.

Areas requiring particular attention of the Task Force included:

## 1. Care and Treatment Continuity

While treatment continuity is a priority in most clinical trials, the urgency is heightened in tuberculosis trials due to the nature of the disease. Treatment interruption is associated with poor outcomes and development of further drug resistance [7]. The long treatment regimens required for tuberculosis complicate efforts to prevent interruptions under normal conditions, let alone during a pandemic. For the small number of participants still receiving an injectable agent, the potential challenges are even greater in terms of travel required to receive their treatment.

Most STREAM trial sites are now under various levels of ‘stay at home’ orders which discourage or prevent movement of participants to trial sites for follow-up visits, including collection of medicine (Table 1). Even where country policies allow travel for medical reasons, this may require a government-issued permit for internal movement and lack of available public transportation may still prevent participants from attending clinics. Furthermore, limiting participant exposure to the health system and potential coronavirus exposure is an important consideration.

These factors require clinical trial sponsors to balance the need for routine treatment management against the possible negative impacts of participant travel to clinics. In the STREAM trial, arrangements have been made for delivery of medicines to the homes of participants who consent and, where necessary, nearby health centers have also been mobilized for administration of injectable agents. To prevent risks to treatment continuity should circumstances further restrict clinic visits or delivery of supplies, the amount of drug provided has been increased. To date, adequate supplies of medicines have been assured for all participants. Video communication has been introduced at most trial sites and has proven feasible for most participants. This enhanced communication supports both treatment adherence, as well as safety monitoring (as described below).

## 2. Supply Chain

Supply chain disruptions also pose a potentially significant challenge during a pandemic. As borders close to travel, including, at times, cargo deliveries, replenishment of medicine stocks can be disrupted. Even domestic travel disruption can threaten continuity of trial supplies where a central depot is utilized in larger countries. The situation can be aggravated if expiration dates of medicines are relatively short and/or by cumbersome importation requirements because frequent replenishments are all the more challenging when timelines for importation are extended. Depending on the duration of restrictions that prevent/extend timelines for importation of medicines, alternative options for medicine procurement, including (where possible) local procurement and borrowing from other local sources (e.g., the National Tuberculosis Program) must be explored.

Supply chain interruptions of laboratory and other supplies (e.g., materials for medicine re-packaging and kits for central laboratory testing) may also occur but pose less serious risks to the trial.

## 3. Participant Safety Monitoring (Including Export of Safety and Microbiology Samples)

Participant safety is always paramount. However, under current conditions, safety monitoring may be interrupted. This is because the risk of exposure to coronavirus in transit to or at health centers may outweigh the monitoring benefits of in-person clinic visits or because local restrictions on movement may prevent participants from attending scheduled clinic visits, or both. Furthermore, restrictions on the export of blood samples can make it necessary to conduct safety testing locally, rather than at central laboratories, and some local laboratories may not have the capacity to conduct all protocol safety assessments. Comprehensive review of local laboratory capacity has been limited by the rapid timeline of transition from central to local laboratories. In most instances, sites have transitioned to their own institution laboratories that routinely serve the tuberculosis programs. Accreditation has been ensured for external private laboratories when utilized for trial samples. Export restrictions may also interrupt transport of microbiology samples for central testing, but these do not carry immediate risk, since microbiology for immediate participant management is already performed locally.

In the STREAM trial, sites are conducting frequent, remote monitoring by phone, including video monitoring wherever possible. Where more intensive monitoring is required for participants identified at greater risk, e.g., ECG monitoring for participants with a previously identified QT prolongation, arrangements have been made to transport participants to the site for necessary assessments and interventions to ensure safety, including local blood analysis. Sputum collection continues wherever possible either during in-person clinic visits or through home collection during the treatment and the immediate follow-up period, as these results may impact on optimal treatment management for participants. Sputum collection in the later follow-up period is generally being rescheduled (provided participants are well) to reduce COVID risks for participants and staff.

The trial continues to store blood and microbiology specimens for future export, provided storage and stability requirements can be met.

Accurate diagnosis of TB patients with respiratory symptoms against the backdrop of other respiratory illnesses, such as COVID-19, can present a clinical challenge. Fortunately, most participants in STREAM have completed, or are approaching the end of, the intensive phase of treatment and TB-related respiratory symptoms should be limited to the small number of participants experiencing treatment failure. Participants presenting with respiratory symptoms will be assessed for both TB for COVID-19 in accordance with local guidelines and testing capacity. Any suspected or confirmed COVID-19 cases will be managed according to local guidelines and documented in a manner similar to other concurrent illnesses in the trial. Early reports of greater severity of COVID-19 illness amongst TB patients [8] heighten the need to prevent exposure of trial participants to the greatest extent possible.

Additional challenges related to trial implementation include: ensuring sufficient human resources at sites when local staff reassignment to COVID-related tasks is occurring and/or staff are personally impacted by COVID-19, continuing to conduct source data verification (SDV) when on-site monitoring by external partners cannot take place, and disruption of community engagement (CE) activities when in-person meetings are not possible.

The experience of the STREAM trial against the backdrop of the pandemic highlights the importance of contingency planning and risk mitigation both before and during unanticipated crises. While some of the challenges highlighted are specific to clinical trial implementation, many issues and potential solutions presented apply equally to operational research of MDR-TB regimens. Guidance from regulatory agencies has been especially useful to clarify acceptable responses to the very real challenges arising out of the pandemic [9,10]. Careful documentation of protocol deviations related to the pandemic is essential and these deviations will be reported to local and global ethics committees, as well as ultimately to regulatory authorities. The trial team will continue to closely monitor the status of the pandemic across trial sites and adapt accordingly to ensure the integrity of the trial, as well as the safety of all participants and site staff.

## Figures and Tables

**Table 1:** STREAM Stage 2 countries and current status of participants and restrictions on travel and import/export.

Country	Participants on Treatment	Participants in Follow Up	Movement Restrictions Preventing Clinic Visits	Restrictions on Import/Export
Ethiopia	Yes	Yes	No	Yes
Georgia	No	Yes	No	No
India	Yes	Yes	Yes	Yes
Moldova	Yes	Yes	No	No
Mongolia	Yes	Yes	No	Yes
South Africa	No	Yes	Yes	Yes
Uganda	Yes	Yes	Yes	Yes

as of 15 April 2020.
